# Identification and Functional Prediction of Long Non-Coding RNA in Longissimus Dorsi Muscle of Queshan Black and Large White Pigs

**DOI:** 10.3390/genes14010197

**Published:** 2023-01-12

**Authors:** Yaqing Dou, Kunlong Qi, Yingke Liu, Chenlei Li, Chenglei Song, Yilin Wei, Zhe Zhang, Xinjian Li, Kejun Wang, Xiuling Li, Ruimin Qiao, Feng Yang, Xuelei Han

**Affiliations:** College of Animal Science and Technology, Henan Agricultural University, Zhengzhou 450002, China

**Keywords:** LncRNAs, intramuscular fat, large white pigs, queshan black pigs, pork quality

## Abstract

Long non-coding RNA (lncRNA) participates in the regulation of various biological processes, but its function and characteristics in intramuscular fat (IMF) deposition in different breeds of pigs have not been fully understood. IMF content is one of the important factors affecting pork quality. In the present study, the differentially expressed lncRNAs (DE lncRNAs) and their target genes were screened by comparing Queshan Black (QS) and Large White (LW) pigs based on RNA-seq. The results displayed 55 DE lncRNAs between QS and LW, 29 upregulated and 26 downregulated, with 172 co-located target genes, and 6203 co-expressed target genes. The results of GO and KEGG analysis showed that the target genes of DE lncRNAs were involved in multiple pathways related to lipogenesis and lipid metabolism, such as the lipid biosynthetic process, protein phosphorylation, activation of MAPK activity, and the Jak-STAT signaling pathway. By constructing regulatory networks, lincRNA-ZFP42-*ACTC1*, lincRNA-AMY2-*STAT1*, and/or lincRNA-AMY2/miR-204/*STAT1* were sieved, and the results indicate that lncRNA could participate in IMF deposition through direct regulation or ceRNA. These findings provide a basis for analyzing the molecular mechanism of IMF deposition in pigs and lay a foundation for developing and utilizing high-quality resources of local pig breeds.

## 1. Introduction

Pork is a crucial part of people’s diet. With the improvement in quality of life and changing dietary preferences, people introduce higher requirements for pork meat quality, nutrient content, and taste. Intramuscular fat (IMF) is a vital trait that determines meat quality; its content is positively correlated with meat quality, and it is one of the key indexes to determine meat quality [1]. Meanwhile, IMF is also an important sensory quality of pork, which will affect the taste and flavor of pork and consumer preference [2]. Producing high-quality meat with high IMF content has always been a major challenge in China’s livestock production. Considering the different genetic backgrounds and artificial breeding methods, the IMF content of Chinese native pigs and western pigs are quite different [3]. In comparison with the Large White (LW) pig breed, which is the most widespread representative western lean pig breed, the Queshan Black (QS) pig, as a local excellent pig breed in Henan Province, has high IMF content, strong fertility, strong adaptability, and excellent meat quality [4,5]. Therefore, these two pig breeds are good animal models for the study of IMF and lay a foundation for the analysis of the molecular regulatory mechanisms that affect fat deposition in pigs.

lncRNAs are RNA transcripts larger than 200 nucleotides in length and with no coding ability that were previously considered transcriptional noises without biological function [6]. However, with the continuous progress in and application of high-throughput sequencing technology, lncRNAs have been found to have important functions in animal fat deposition and other processes [7,8]. In recent years, research on the regulatory role of lncRNAs in fat deposition and sarcomere improvement has been increasing, and IMF deposition is influenced by several genes and signaling pathways. SRA is the first lncRNA that regulates adipocytes and is highly expressed in adipocytes. It enhances its transcriptional activity by binding to *PPARγ*, thus promoting adipocyte differentiation [9]. Studies have shown that *KLF6* knockdown may affect the formation and lipid metabolism of bovine adipose cells through various biological and molecular pathways such as calcium and cAMP signaling pathways [10]. *C/EBPα* is a key transcriptional regulator of adipogenesis, and it promotes adipogenesis by affecting the H3K4me3 and H3K27me3 histone modifications of the *C/EBPα* locus and activating cis-*C/EBPα* transcription [11]. PU.1 overexpression in preadipocytes downregulates *PPARγ* and *C/EBPα*, thus inhibiting adipogenesis, and the PU.1 antisense strand can transcribe the un-encodable transcript PU.1 AS lncRNA, which combines with PU.1 mRNA to form a complex mRNA/AS lncRNA, hinting at the translation of PU.1 and promoting adipogenesis [12,13]. LncIMF4 promotes adipogenesis in porcine intramuscular preadipocytes by attenuating autophagy and inhibiting lipolysis [14]. By selecting three pairs of full-sib LW × Min F2 sows with different IMF content for RNA-seq, IRLnc further affected IMF decomposition by regulating the expression of IMF [15]. After analyzing RNA-seq data from Laiwu and LW pigs, Huang et al. found that 55 lncRNAs and 513 mRNAs were differentially expressed; through cis and trans regulation analysis and co-expression network construction, five key lncRNAs and their target genes were finally identified, and these structures may play a key regulatory role in fat accumulation and differentiation [16]. The analysis of lncRNA expression levels in the IMF of Landrace and Jinhua pigs showed that 119 lncRNAs were differentially expressed, and after co-expression with mRNAs, 6 of the co-expressed lncRNAs were associated with lipid metabolism and fat deposition-related pathways, thus providing a basis for subsequent studies on fat deposition [17]. LncIMF2 can sponge for miR-217, thus influencing the expression level of miR-217 target genes and promoting the proliferation and differentiation of porcine intramuscular precursor adipocytes [18]. LncRNA-FNIP2 accelerates lipid synthesis through the lncRNA-FNIP2/miR-24-3p/*FNIP2* axis in chicken abdominal adipocytes [19]. LncRNA regulates adipogenesis through multiple mechanisms, and it is a regulator that affects adipocyte differentiation [20,21].

Pigs are highly similar to humans in anatomy, physiology, and genome, making them ideal animal models for biomedical research [22]. Therefore, the molecular regulation mechanism of fat deposition in pigs should be explored. The function of lncRNA target genes is the starting point for the study of lncRNA function. Wang et al. identified 17 DE lncRNAs and 180 mRNAs in Laiwu black pigs with different IMF content, revealing seven candidate genes related to IMF accumulation, which is critical for further understanding of the molecular mechanism controlling adipogenesis [23]. In the present study, the longissimus dorsi (LD) of QS and LW pigs were used for RNA-seq, from which lncRNAs and mRNAs that are differentially expressed between the two breeds were extracted. Further, through target gene prediction and functional enrichment analysis of lncRNAs, the key lncRNAs and their target genes that regulate pig fat deposition were screened. At the same time, the miRNAs combined with key lncRNAs were predicted to construct the ceRNA regulatory network. This study aims to explore the molecular regulation mechanism that affects pig IMF deposition and provide a theoretical basis for further analysis of the pig fat deposition regulation network.

## 2. Materials and Methods

### 2.1. Animals and Tissue Preparation

The QS and LW pigs used in this experiment were obtained from Henan Fenghua Breeding Share Limited Company and were fed under the same feeding and management conditions. Three QS and three LW with a weight of 100 kg were selected and fasted for 24 h. After slaughtering, LD samples at the 6th to 7th ribs on the right were collected for RNA extraction and placed in liquid nitrogen immediately. Then, all samples were stored at −80 °C for further analysis.

### 2.2. RNA Sequencing, Quality Control and Mapping

Total RNA was isolated using TRIzol reagent (15596026, Thermo Fisher Scientific, Waltham, MA, USA) according to the manufacturer’s instructions. RNA purity was checked using a NanoPhotometer^®^ spectrophotometer (IMPLEN, Westlake Village, CA, USA). RNA quality detection, RNA sequencing, quality control, and mapping have been described in detail in our previous publication [5]. The reference genome and gene model annotation files can be downloaded directly from the genome website. The reference genome was indexed using HISAT2 v.2.0.4, and paired-end clean reads were aligned to the reference genome by using HISAT2 v.2.0.4 [24]. HISAT2 was run with ‘--RNA-strandness RF’ and the defaults for other parameters. The mapped reads of each sample were assembled by StringTie (v.1.3.3) [25].

### 2.3. Coding Potential Analysis

Known transcripts and transcripts less than 200 bp in length were filtered out. We determined whether the encoding potential was the key condition to determine lncRNAs. CNCI (v.2), CPC2 (v.0.1), and Pfam-sca (v.1.3) were used to screen the transcripts, and transcripts predicted with coding potential by either/all of the three tools mentioned above were filtered out [26,27,28]. Transcripts with no coding potential were considered as candidate lncRNAs. The transcripts considered to have coding potential by at least one piece of coding potential software are TUCP (transcripts of uncertain coding potential). They have high evolutionary conservation but may include short open reading frames (ORFs). They can encode proteins and may serve either as lncRNAs or as small peptides.

### 2.4. Quantification and Differential Expression Analysis

StringTie (v.2.1.1) was used to calculate the fragments per kilobase of transcript per million mapped reads (FPKMs) of both lncRNAs and coding transcripts in each sample [25]. The Ballgown suite includes functions for the interactive exploration of the transcriptome assembly, visualization of transcript structures and feature-specific abundances for each locus, and post-annotation from assembly features to annotated features [29]. Cuffdiff provides statistical routines for determining differential expression in digital transcript or transcript expression data by using a model based on the negative binomial distribution [30]. The differentially expressed lncRNAs, miRNAs, and mRNAs were screened using the DESeq2 R package (v.1.10.1) that satisfied the condition of *p* < 0.05 and |log2 fold change| ≥ 1.

### 2.5. Prediction of lncRNA Target Genes

In this study, the co-expression and co-localization genes of lncRNA were predicted to explore the functions of lncRNAs. We retrieved the genes 100 kb upstream and downstream of lncRNAs and analyzed their functions. “Trans-acting” refers to the co-expression relationship between lncRNA and mRNA. To explore the trans roles of these molecules, we used a custom script to calculate the Pearson’s coefficient between the lncRNAs and coding genes. The correlation coefficient was greater than 0.95 to predict lncRNA target genes.

### 2.6. Analysis of Enrichment

GO enrichment analysis of the related genes of DE lncRNAs was performed using KOBAS. GO terms with a corrected *p*-value of less than 0.05 were considered to be significantly enriched in differential expressed genes. KEGG is a database resource for understanding the advanced capabilities and utilities of biological systems [31]. We performed statistical enrichment of differentially expressed genes or lncRNAs target genes in the KEGG pathways using KOBAS software [32]. The results were visualized using the ClueGo (v.2.5.8) and CluePedia plugins (v.1.5.8) in Cytoscape software [33]. Gene set enrichment analysis (GSEA) was performed using the OmicStudio tools at https://www.omicstudio.cn/tool (accessed on 19 November 2022), The pathway with |NES| > 1, NOM *p*-val < 0.05, FDR *q*-val < 0.25 was considered statistically significant.

### 2.7. Regulatory Networks Construction

DE lncRNAs and their co-expressed target mRNAs which may play a role in the regulation of intramuscular fat deposition were screened, and the interactions between miRNAs and DE lncRNAs and mRNAs were analyzed using miRanda software [34] to construct lncRNA-mRNA and lncRNA-miRNA-mRNA regulatory networks and were visualized using Cytoscape (v.3.9.1).

### 2.8. Protein Interaction Network Analysis

The differentially expressed genes were subjected to protein–protein interaction (PPI) analysis based on the STRING database, which is known and predicted for PPIs. The screened differential genes were imported into STRING for online analysis, and the corresponding data were imported into Cytoscape (v.3.9.1) for MCODE (v.2.0.0) analysis. The parameters were set as degree cutoff = 2, node score cutoff = 0.2, k core = 2, maximum depth = 100, and the score was set to ≥4 to obtain the two closest clusters from the PPI.

### 2.9. Verification of Sequencing Data

We randomly selected five lncRNAs and five mRNAs to validate their expression. Approximately 1 μg of each RNA sample was used in the PrimeScript™ RT reagent kit with gDNA Eraser (Perfect Real Time, Code No. RR047A, Takara, Beijing, China) to convert the total RNA to cDNA. Next, qRT-PCR was performed using TB Green^®^ Premix Ex Taq™ II (Code No. RR820A, Takara, Beijing, China) on the CFX96 Real-Time PCR Detection System (Bio-Rad, Hercules, CA, USA). Glyceraldehyde3-phosphate dehydrogenase (*GAPDH*) was used as the normalization control, and all reactions were carried out in triplicate. The 2^−ΔΔCT^ method was used to quantify relative expression levels.

## 3. Results

### 3.1. Identification and Genomic Characterization of lncRNAs and TUCPs

**Figure 1:**
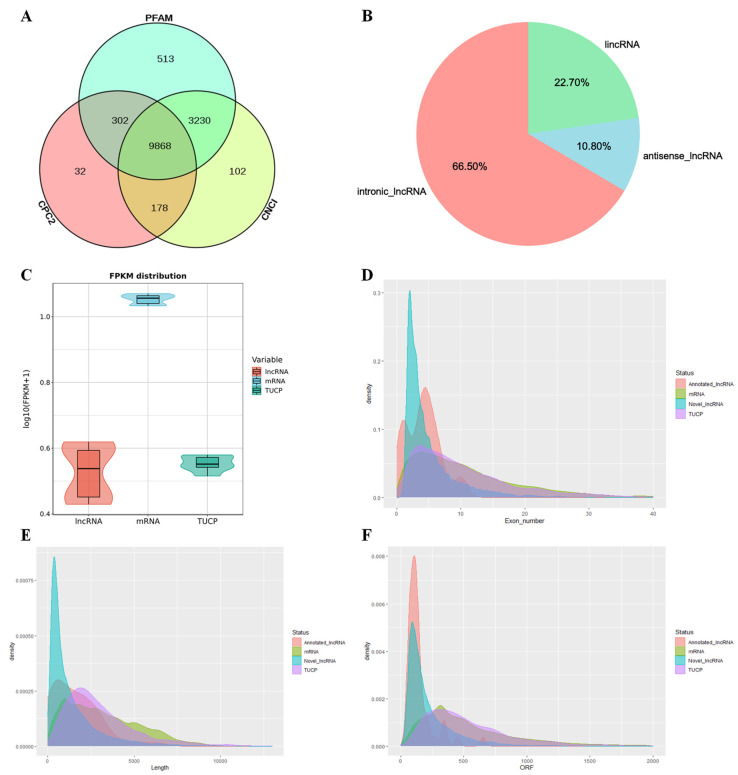
Identification and genomic characterization of lncRNAs and TUCPs. (**A**) Screening of candidate lncRNAs in porcine LD muscles; (**B**) Classification of candidate lncRNA; (**C**) Violin plot of the expression level for lncRNA, TUCP, and mRNA transcripts; (**D**–**F**) Density distribution of lncRNA, TUCP, and mRNA transcripts, (**D**) Exon number; (**E**) Length; (**F**) Length of open reading frame.

In this study, we obtained the raw reads by RNA-seq and filtered out articulated, null, and low-quality sequences. Clean reads obtained in each library accounted for 94.33%–95.20% of the raw reads [5]. A total of 9918 lncRNAs, 4415 TUCPs, and 10,107 mRNAs were identified from the two groups of LD muscle. Among these lncRNAs, 50 were annotated lncRNAs, and 9868 were novel lncRNAs with proportions of 22.7% lincRNAs (2240), 10.8% antisense lncRNAs (1070), and 66.5% intronic lncRNAs (6561, Figure 1A,B). The expression levels of mRNAs were higher than those of lncRNAs and TUCPs in both pig breeds (Figure 1C). The average exon number of mRNAs was approximately 12, while the average exon number of novel lncRNAs, annotated lncRNAs, and TUCPs were approximately 5, 4, and 12, respectively (Figure 1D). The average length of TUCPs was 3531.129 nt, and the average length of mRNAs was 3296.133 nt, which was longer than the average length of novel lncRNAs and annotated lncRNAs (1580.563 nt/1650.96 nt, Figure 1E). The ORF of TUCPs was longer than that of the mRNAs and lncRNAs. TUCPs had an average length of 622.8725 nt, while the average length of novel lncRNAs, annotated lncRNAs, and mRNAs were 231.8486 nt, 147.12 nt, and 593.891 nt, respectively (Figure 1F). Overall, compared with mRNAs, lncRNAs are characterized by a lower number of exons, shorter length, shorter average length of ORFs, and lower expression levels.

### 3.2. Differentially Expressed lncRNAs/TUCPs in the LD Muscle of QS and LW Pigs

**Figure 2:**
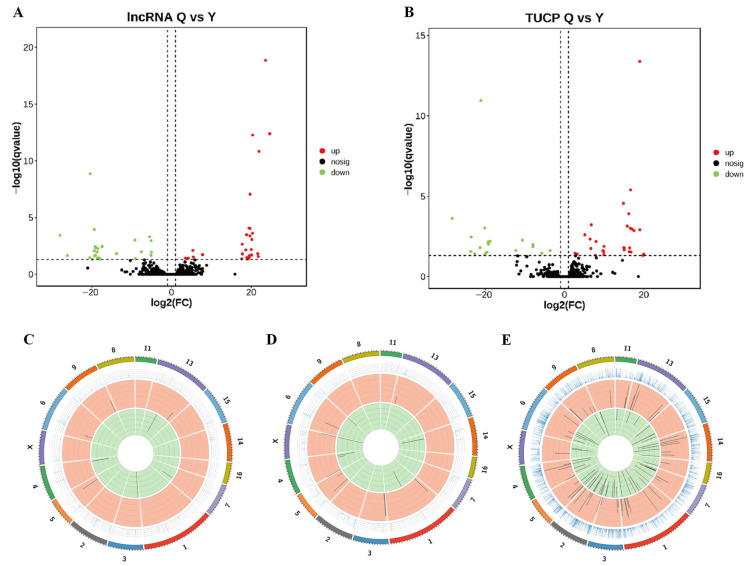
Difference in lncRNAs/TUCPs expression in LD muscle of QS and LW pigs. (**A**) Volcano plots of the DE-lncRNAs; (**B**) Volcano plots of the DE-TUCPs; (**C**) Chromosome distribution of differentially expressed lncRNAs; (**D**) TUCPs; (**E**) mRNAs. Red points represent upregulated lncRNAs/TUCPs, while green points represent downregulated lncRNAs/TUCPs.

Of the 55 DE lncRNAs detected, 29 were upregulated, and 26 were downregulated (Figure 2A). A total of 46 TUCPs were differentially expressed, 28 upregulated and 18 downregulated (Figure 2B). We calculated the total mapped reads density for each chromosome (forward and reverse strands) in the genome and found that the upregulated DE lncRNAs were mainly distributed on chromosomes 5, 6, 9, and 13, while the downregulated DE lncRNAs were mainly located on chromosomes 3, 4, 7, and 13 (Figure 2C). DE TUCPs were mainly concentrated on chromosomes 1, 3, 4, 5, 6, 13, 14, and X, among which chromosomes 3 and 5 had more upregulated TUCPs, but chromosome 4 had more downregulated TUCPs compared with other chromosomes (Figure 2D). DE mRNAs were mainly distributed on chromosomes 1, 2, 3, 4, 5, 6, 7, 8, 9, 11, 13, 14, 15, 16, and X. Chromosomes 1, 2, 13, and 14 had more upregulated mRNAs, and chromosomes 1, 2, and 7 had more downregulated mRNAs (Figure 2E).

### 3.3. Comprehensive Analysis of DE lncRNAs and Targeted mRNAs

**Figure 3:**
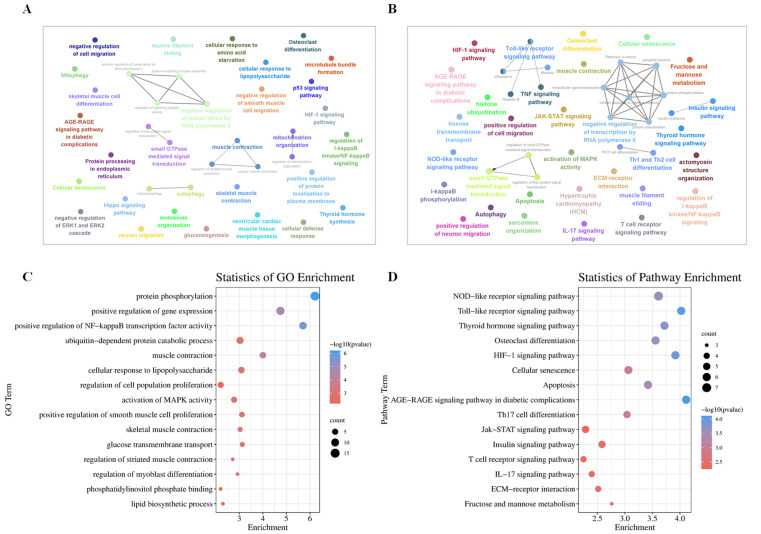
GO and KEGG analysis of DE lncRNAs co-location and co-expression of targeted DE mRNAs. (**A**) ClueGO network of DE lncRNAs co-location of targeted DE mRNAs; (**B**) ClueGO network of DE lncRNAs co-expression of targeted DE mRNAs; (**C**) GO biological processes and (**D**) KEGG analysis of DE lncRNAs co-expression of targeted DE mRNAs. Each node represents a term.

In order to understand the potential functions of novel lncRNAs, we conducted co-localization and co-expression analysis of candidate DE lncRNAs. Among the 55 DE lncRNAs, the co-localization analysis revealed 45 DE lncRNAs corresponding to 172 related mRNAs, and the co-expression analysis revealed 55 DE lncRNAs corresponding to 6203 related mRNAs. Further analysis showed that 7 DE lncRNAs were co-located with 8 DE mRNAs, and 55 DE lncRNAs were co-expressed with 326 DE mRNAs. It was obtained by enrichment analysis that the DE target genes co-localized with DE lncRNAs were mainly involved in muscle contraction regulation, cell differentiation, thyroid hormone synthesis, and cellular response to the lipopolysaccharide and p53 signaling pathway (Figure 3A). The DE target genes co-expressed with DE lncRNAs were mainly involved in the activation of MAPK activity, protein phosphorylation, the insulin signaling pathway, and fructose and mannose metabolism (Figure 3B).

GO analysis of co-localized DE target genes showed that, skeletal muscle contraction, apolipoprotein binding, calcium channel regulator activity, skeletal muscle cell differentiation, regulation of I-kappaB kinase/NF-kappaB signaling, gluconeogenesis, muscle contraction, and other items were significantly enriched (Table S1). The results of KEGG analysis showed that 17 pathways such as cellular senescence, the HIF-1 signaling pathway, thyroid hormone synthesis, the AGE-RAGE signaling pathway in diabetic complications, and the osteoclast differentiation pathways were significantly enriched (Table S2).

GO enrichment analysis of co-expressed DE target genes showed that the main enrichments were positive regulation of gene expression, muscle contraction, cellular response to lipopolysaccharide, skeletal muscle contraction, regulation of myoblast differentiation, lipid biosynthetic process, regulation of cell population proliferation, phosphatidylinositol phosphate binding, and other terms (Figure 3C, Table S3). The results of KEGG analysis showed that osteoclast differentiation, the Jak-STAT signaling pathway, Th17 cell differentiation, the thyroid hormone signaling pathway, ECM-receptor interaction, the insulin signaling pathway, apoptosis, and the T cell receptor signaling pathway were significantly enriched (Figure 3D, Table S4).

**Figure 4:**
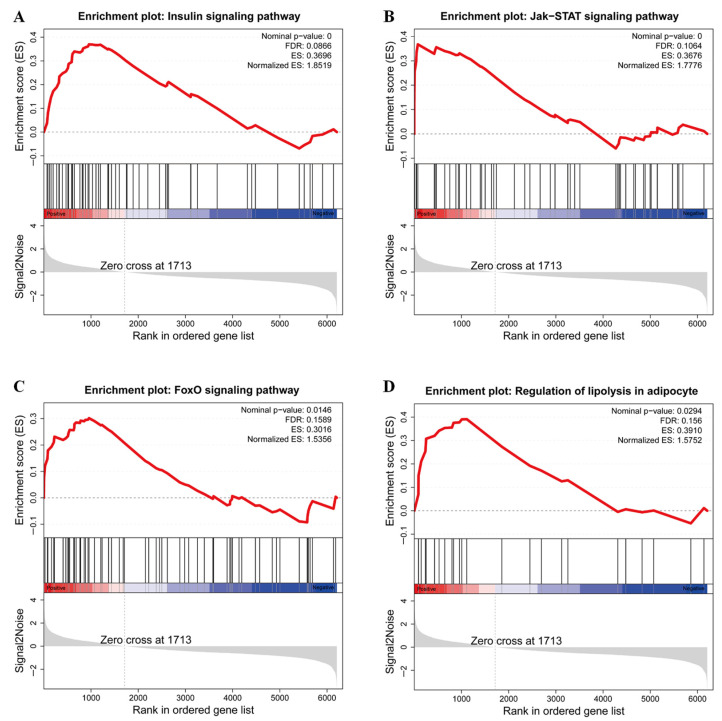
Gene set enrichment analysis (GSEA) indicated significant enrichment in (**A**) Insulin signaling pathway; (**B**) Jak-STAT signaling pathway; (**C**) FoxO signaling pathway and (**D**) Regulation of lipolysis adipocyte.

Gene Set Enrichment Analysis (GSEA) of DE lncRNAs co-expressed target genes showed that the insulin signaling pathway, FoxO signaling pathway, and Jak-STAT signaling pathway were also highly enriched (Figure 4A–C), which was consistent with the results of KEGG enrichment analysis. In addition, the regulation of lipolysis adipocyte was also significantly enriched (Figure 4D).

### 3.4. LncRNA-mRNA and lncRNA-miRNA-mRNA Interaction Network Analysis

**Figure 5:**
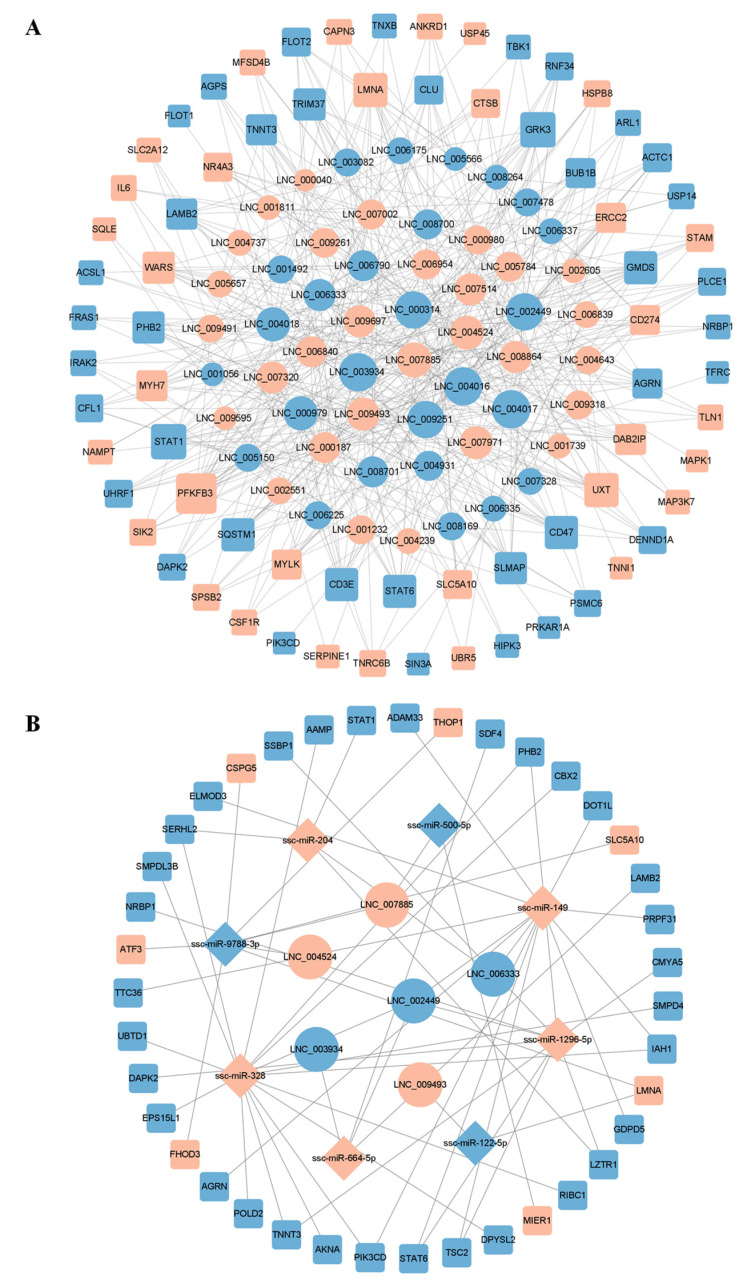
Construction of the potential lncRNA-mRNA and lncRNA-miRNA-mRNA regulatory network. (**A**) LncRNA-mRNA co-expression network; (**B**) Analysis of the lncRNA-miRNA-mRNA network. Circular nodes indicate lncRNAs, diamond nodes indicate miRNAs, square nodes indicate mRNAs, pink nodes indicate upregulated transcripts, and blue nodes indicate downregulated transcripts.

The lncRNA-mRNA co-expression network consisted of 126 network nodes with 427 junctions between 55 DE lncRNAs and 71 DE mRNAs (Figure 5A). The central nodes are LNC_003934, LNC_000314, LNC_002449, LNC_007885, LNC_009493, LNC_006333, and LNC_004524, and the corresponding target genes are *LZTR1*, *CFL1*, *AGRN*, *STAT6*, *ZNF148*, *STAT1*, *LMNA*, and so on. These DE lncRNAs and mRNAs may be potential key genes for the regulation of IMF deposition. Each lncRNA can be associated with one or more mRNAs, such as LNC_000040-*ERCC2*, LNC_003934-*ACTC1*, *AGRN*, and *LMNA*. Each mRNA can also be associated with one or more lncRNAs, such as *MAPK1*-LNC_009261, *ACTC1*-LNC_002449, LNC_000314, and LNC_003934.

The constructed DE lncRNA-miRNA-mRNA ceRNA regulatory network included 6 DE lncRNAs, 8 DE miRNAs, and 39 DE mRNAs, as shown in Figure 5B. DE mRNAs such as *STAT1*, *STAT6*, *AGRN*, *LMNA*, and *PIK3CD* were the predicted target genes of miRNA. One lncRNA could bind to one or more miRNAs, and each miRNA could bind to one or more lncRNAs, where LNC_006333 adsorbed both ssc-miR-149 and ssc-miR-204, constituting the LNC_006333/ssc-miR-149/*STAT6* and LNC_006333/ssc-miR-204/*STAT1* regulatory networks.

### 3.5. Network Analysis of DE Target Genes

**Figure 6:**
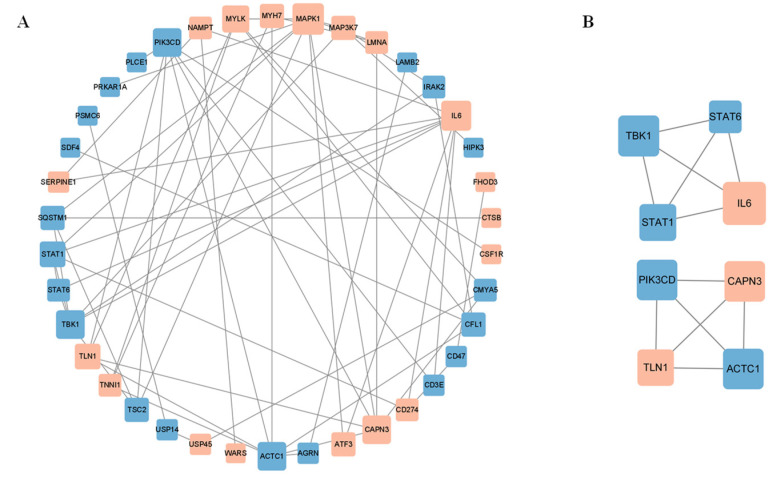
Construction of protein-protein interaction (PPI) network. (**A**) PPI network of differentially expressed target genes; (**B**) MCODE analysis of differentially expressed target genes, module 1 is pictured above, while module 2 is shown below.

The DE mRNAs in the above regulatory networks were analyzed using STRING and Cytoscape software to obtain the PPI network (Figure 6A). Two clustering modules were obtained using the MCODE plug-in, each containing four nodes and six contigs. Module 1 consists of *STAT1*, *STAT6*, *IL6*, and *TBK1*, where *IL6* is the core gene. Module 2 consists of *ACTC1*, *PIK3CD*, *TLN1*, and *CAPN3*, where *ACTC1* is the core gene (Figure 6B).

### 3.6. Validation of Gene Expression in RNA-seq

**Figure 7:**
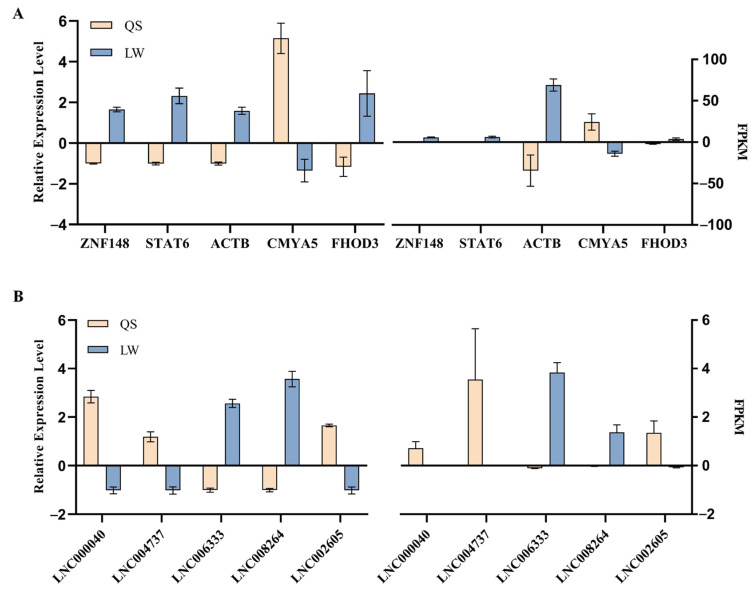
Validation of gene expression in RNA-seq. (**A**) Verification of mRNA expression level; (**B**) Verification of lncRNA expression level. qRT-PCR results are shown on the left and RNA-seq results are shown on the right. The accuracy of the sequencing results was verified by qRT-PCR. The data represent the mean ± SEM from three biological replicates, and each measurement was repeated at least thrice.

To further validate the RNA-seq results and detect the expression levels, we randomly selected five DE lncRNAs and five DE mRNAs for qRT-PCR. The experimental results were consistent with RNA-seq (Figure 7), revealing that the gene expression trends detected by these two methods are consistent. Therefore, the RNA-seq data are highly reliable and accurate. Details of primers used for real-time PCR amplification are shown in Table S5.

## 4. Discussion

Fat deposition is a complex process, which involves biochemical processes such as proliferation, differentiation, occurrence, and apoptosis of adipocytes. IMF participates in several biological pathways such as hormones, muscle development, and fat deposition, which are affected by breed, nutrition, and deposition time. It is an important index for the evaluation of meat quality. lncRNAs can bind to nucleic acids and proteins and regulate gene expression through various mechanisms, and play a complex regulatory role in animal growth and development. In addition, lncRNAs can also act as signals to stimulate or repress transcriptional processes, act as epigenetic regulators, and even play the role of scaffold to interact with other proteins to produce ribonucleoprotein complexes. However, the functions and characteristics of lncRNAs in porcine fat deposition and metabolism are poorly understood, and the regulatory mechanisms have not been fully elucidated and need to be further explored.

In this study, the expression level of lncRNAs in the LD of QS and LW pigs was analyzed by RNA-seq, and 55 DE lncRNAs were identified. lncRNAs have no coding ability and can play a role by binding to proteins or chromosomes, especially transcription factors [35]. Huang et al. found by RNA-seq that NDUFC2-AS lncRNA in bovine adipose tissue promoted lipid differentiation by upregulating the expression levels of *THRSP* and *C/EBP-α* in buffalo [36]. It was found that the highly expressed lnc-LLMA interacted with *MTTP* and *GYS2* in Duroc pigs, affecting the process of fat decomposition and transport [37]. lncRNA Blnc1 is a novel nuclear lncRNA, *EBF2* acts as its target gene, and these two structures combine to form a nucleoprotein complex, which promotes beige and brown adipocyte differentiation [38]. By constructing a co-expression network between lncRNAs and mRNAs, Zheng et al. found that lncRNAs *AC004797.1*, *PRKG1-AS1*, and *GRPC5D-AS1* may be associated with aging-associated muscle atrophy [39]. In addition, lncRNAs can adsorb miRNAs and act as ceRNA to regulate the expression of their target genes. lncRNA ADNCR is a downregulated lncRNA during bovine adipocyte differentiation, inhibits adipocyte differentiation by adsorbing miR-204, and affects the expression level of miR-204 target gene *SIRT1* [40]. FDNCR1 competitively binds to miR-204, which inhibits the expression of the transforming growth factor β receptor 1 (*TGFBR1*) gene, to regulate porcine precursor adipocyte differentiation [41]. MSTRG.25116.1 is progressively upregulated during chicken abdominal adipocyte differentiation and directly regulates fatty acid amide hydrolase (*FAAH*) gene transcriptional activity in a trans-regulatory manner, while MSTRG.25116.1 was found to also act as a molecular sponge that competitively binds to gga-miR-1635 to promote the post-transcriptional expression of *FAAH* and participates in the regulation of chicken fat formation [42]. *SYISL*, a conserved lncRNA in pigs and humans, can act as a sponge for miR-23a-3p, miR-103-3p, and miR-205-5p to increase the expression of the muscle atrophy inducer genes *FoxO3a*, *MuRF1* and Atrogin-1, thus participating in the cross-species muscle atrophy process [43]. Therefore, in the present study, we analyzed the potential functions of DE lncRNAs by co-location and co-expression of related genes and predicted the miRNAs bound by the screened DE lncRNAs to construct ceRNA regulatory networks for further functional mining of lncRNAs.

In order to further explore the potential functions of DE lncRNAs in pork quality regulation such as IMF deposition and muscle growth and development, we analyzed the intersection of DE mRNAs and target genes of DE lncRNAs by GO and KEGG enrichment analysis. GO results showed that DE lncRNAs are involved in muscle contraction, response to muscle activity, skeletal muscle cell differentiation, sarcomere organization, and myofibril development. Muscle consists of muscle fibers, which serve as the material basis for meat quality traits, and its type is closely related to meat quality, which is an important parameter for the evaluation of meat quality [44,45]. Studies have shown that lncRNA is a participant in the regulation of muscle fiber types [46]. For example, linc-MYH gene deletion leads to phenotypic phenomena such as muscle hypertrophy and muscle weight gain in mice, which may be related to the mechanism of muscle atrophy [47]. Therefore, DE lncRNAs in this study may also play an important regulatory role in muscle growth and development. They are also involved in the activation of MAPK activity, protein phosphorylation, glucose transmembrane transport, lipid droplet formation, and lipid biosynthetic process, and the MAPK signaling pathway plays an important role in insulin resistance, T2DM, and inflammation [48]. Insulin promotes glucose uptake and conversion to glycerol 3 phosphate, thus promoting lipid synthesis and storage. The higher the level of protein phosphorylation, the faster the rate of glycolysis [49], while regulating the rate of pH decrease improves meat quality by affecting protein hydrolysis [50]. *MAPK1*, *IL6,* and *PIK3CD* are involved in the significantly enriched terms above and are associated with lipid metabolism, muscle development, inflammatory response, and cell proliferation migration, respectively [51,52,53]. Therefore, the corresponding lncRNAs may be a potential factor in meat quality regulation. Based on KEGG analysis, the thyroid hormone synthesis pathway, p53 signaling pathway, fructose and mannose metabolism, and ECM-receptor interaction were significantly enriched. Thyroid hormones are important regulators of lipid metabolism and are involved in the regulation of multiple metabolic pathways, including lipid synthesis and lipid oxidation [54]. ECM–receptor interaction affects meat quality by altering IMF content by influencing intramuscular adipocyte differentiation and lipid anabolism [55]. GSEA results reconfirmed the KEGG pathway analysis results, the Jak-STAT signaling pathway, FoxO signaling pathway, insulin signaling pathway, and regulation of lipolysis adipocyte were all highly enriched, suggesting that these pathways have important effects on the process of fat metabolism. Adipocytes play an important role in energy balance, lipid storage, and systemic insulin homeostasis, and the Jak-STAT signaling pathway participates in adipocyte differentiation and mediates cell fate such as apoptosis, differentiation, and proliferation [56,57,58].

In the present study, the construction of the PPI interaction network revealed a high degree of correlation and a strong linkage between four genes, namely, *STAT1*, *STAT6*, *IL6*, and *TBK1*. The STAT family plays a regulatory role in adipogenesis [59,60]. As a member of the STAT family, *STAT1* is related to adipocyte differentiation and affects the process of lipid metabolism [61,62], and plays an important role in the process of muscle regeneration [63]. LNC_006333 is co-expressed with *STAT1* and binds to miR-204, and *STAT1* is the target gene of miR-204 and participates in many pathways related to lipid metabolism. Therefore, LNC_006333 can directly regulate gene expression and exert its biological function through ceRNA, thus affecting the expression level of *STAT6*, *IL6*, and *TBK1* while regulating *STAT1*. LNC_006333 is a lincRNA between *PRMT6* and *AMY2*, and *AMY2* is the nearest protein-coding gene to LNC_006333. Therefore, it is named lincRNA-AMY2, and lincRNA-AMY2-*STAT1* and/or lincRNA-AMY2/miR-204/*STAT1* may be an important regulatory network that affects fat deposition in pigs. In the present study, lncRNAs such as EAD1-AS (LNC_004524), lincRNA-STRIT1 (LNC_002449), HNRNPH3-IT (LNC_003082), and IBA57-IT (LNC_004931) regulate fat deposition in the above manner. *ACTC1* is involved in the regulation of the proliferation cycle of bovine myogenic cells, which is positively correlated with myogenesis and promoting adipocyte differentiation, thus playing a biological function in muscle development and fat deposition [64]. LNC_003934, which is located between *SLC7A2* and *FAT1*, is a lincRNA, named lincRNA-FAT1 because it is the closest to FAT1. It directly regulates *ACTC1* and affects the expression levels of *PIK3CD*, *TLN1*, and *CAPN3*. In addition, lncRNAs such as PLP1-IT (LNC_009595), lincRNA-PPP2R5A (LNC_009318), lincRNA-RORA (LNC_000187), and lincRNA-MIR99A (LNC_002605) regulate the fat deposition process in the same way. In summary, the regulation of different lncRNAs differs. Through a series of analyses, this study screened regulatory networks such as lincRNA-AMY2-*STAT1* and/or lincRNA-AMY2/miR-204/*STAT1*, and lincRNA-FAT1-*ACTC1* may affect IMF deposition by regulating adipocyte proliferation and differentiation and, thus, regulate pork quality. The results of this study provide an important reference for uncovering the regulatory mechanisms of intramuscular fat deposition in pigs and lay the foundation for further understanding the genetic mechanisms of pork quality formation.

## 5. Conclusions

In the present research, based on the RNA-seq of LD muscle tissue of QS and LW pigs, 55 DE lncRNAs were identified, 29 upregulated and 26 downregulated. Through targeted prediction, we discovered that 172 target genes were co-located, and 6203 target genes were co-expressed. Functional enrichment analysis revealed that it was involved in many pathways related to lipid metabolism, such as the activation of MAPK activity, the lipid biosynthetic process, the thyroid hormone signaling pathway, and the Jak-STAT signaling pathway. The regulatory networks of lincRNA-ZFP42-*ACTC1*, lincRNA-AMY2-*STAT1,* and/or lincRNA-AMY2/miR-204/*STAT1* were filtered out, which were potentially related to lipid deposition. These results provide a reference basis for analyzing the mechanism of intramuscular fat deposition in pigs.

## Data Availability

All raw data of high-throughput sequencing have been deposited to the National Genomics Data Center (NGDC, https://bigd.big.ac.cn, accessed on 19 September 2021) with the dataset accession number CRA005025. The data will be available by 19 September 2023.

## Supplementary Materials

The following supporting information can be downloaded at: https://www.mdpi.com/article/10.3390/genes14010197/s1, Table S1: GO biological processes analysis of DE lncRNAs co-location of targeted DE mRNAs (15 terms); Table S2: KEGG analysis of DE lncRNAs co-location of targeted DE mRNAs (15 pathways); Table S3: GO biological processes analysis of DE lncRNAs co-expression of targeted DE mRNAs (15 terms); Table S4: KEGG analysis of DE lncRNAs co-expression of targeted DE mRNAs (15 pathways); Table S5: The primers used for the validation of lncRNAs and mRNAs.
