# Supplementary material for: Identification and Functional Prediction of Long Non-Coding RNA in Longissimus Dorsi Muscle of Queshan Black and Large White Pigs
Source: Genes (Basel). 2023 Jan 12;14(1):197. doi: 10.3390/genes14010197 (PMC9858627; doi:10.3390/genes14010197)
Supplement: Supplementary file 1 [file genes-14-00197-s001.zip › genes-2083409-supplementary.pdf]

Table S1. GO biological processes analysis of DE lncRNAs co-location of targeted DE mRNAs (15 terms).

Term	Database	ID	Input number	Background number	*P*-Value	Corrected *P*-Value	Input
protein binding	Gene Ontology	GO:0005515	7	11779	0.001296	0.019351	*SQSTM1*|*RRAD*|*ZNF148*|*ATF3*|*TNNI1*|*SERPINE1*|*CANX*
regulation of striated muscle contraction	Gene Ontology	GO:0006942	1	8	0.001833	0.019351	*TNNI1*
negative regulation of smooth muscle cell migration	Gene Ontology	GO:0014912	1	13	0.00285	0.019459	*SERPINE1*
apolipoprotein binding	Gene Ontology	GO:0034185	1	16	0.00346	0.019573	*CANX*
skeletal muscle contraction	Gene Ontology	GO:0003009	1	26	0.00549	0.022185	*TNNI1*
regulation of I-kappaB kinase/NF-kappaB signaling	Gene Ontology	GO:0043122	1	29	0.006099	0.022361	*SQSTM1*
negative regulation of endothelial cell apoptotic process	Gene Ontology	GO:2000352	1	31	0.006504	0.022996	*SERPINE1*
calcium channel regulator activity	Gene Ontology	GO:0005246	1	35	0.007314	0.023411	*RRAD*
cardiac muscle contraction	Gene Ontology	GO:0060048	1	44	0.009136	0.024652	*TNNI1*
cellular response to amino acid starvation	Gene Ontology	GO:0034198	1	44	0.009136	0.024652	*ATF3*
skeletal muscle cell differentiation	Gene Ontology	GO:0035914	1	45	0.009338	0.024652	*ATF3*
gluconeogenesis	Gene Ontology	GO:0006094	1	45	0.009338	0.024652	*ATF3*
small GTPase mediated signal transduction	Gene Ontology	GO:0007264	1	94	0.019201	0.03553	*RRAD*
muscle contraction	Gene Ontology	GO:0006936	1	105	0.021403	0.037173	*TNNI1*
cellular response to lipopolysaccharide	Gene Ontology	GO:0071222	1	158	0.031953	0.047569	*SERPINE1*

Table S2. KEGG analysis of DE lncRNAs co-location of targeted DE mRNAs (15 pathways).

Term	Database	ID	Input number	Background number	*P*-Value	Corrected *P*-Value	Input
Cellular senescence	KEGG PATHWAY	hsa04218	2	160	0.000466	0.019351	*SQSTM1*|*SERPINE1*
Mitophagy - animal	KEGG PATHWAY	hsa04137	1	65	0.013374	0.030091	*SQSTM1*
p53 signaling pathway	KEGG PATHWAY	hsa04115	1	72	0.014783	0.031986	*SERPINE1*
Thyroid hormone synthesis	KEGG PATHWAY	hsa04918	1	74	0.015185	0.031986	*CANX*
Antigen processing and presentation	KEGG PATHWAY	hsa04612	1	77	0.015789	0.032564	*CANX*
Complement and coagulation cascades	KEGG PATHWAY	hsa04610	1	79	0.01619	0.032711	*SERPINE1*
AGE-RAGE signaling pathway in diabetic complications	KEGG PATHWAY	hsa04933	1	100	0.020402	0.036393	*SERPINE1*
Chagas disease (American trypanosomiasis)	KEGG PATHWAY	hsa05142	1	103	0.021003	0.036801	*SERPINE1*
HIF-1 signaling pathway	KEGG PATHWAY	hsa04066	1	109	0.022203	0.037897	*SERPINE1*
Osteoclast differentiation	KEGG PATHWAY	hsa04380	1	128	0.025994	0.042164	*SQSTM1*
Apelin signaling pathway	KEGG PATHWAY	hsa04371	1	137	0.027785	0.044011	*SERPINE1*
Fluid shear stress and atherosclerosis	KEGG PATHWAY	hsa05418	1	139	0.028182	0.044248	*SQSTM1*
Phagosome	KEGG PATHWAY	hsa04145	1	152	0.030764	0.047097	*CANX*
Hippo signaling pathway	KEGG PATHWAY	hsa04390	1	154	0.03116	0.047097	*SERPINE1*
Necroptosis	KEGG PATHWAY	hsa04217	1	162	0.032745	0.047772	*SQSTM1*
Protein processing in endoplasmic reticulum	KEGG PATHWAY	hsa04141	1	165	0.033339	0.047772	*CANX*
Human T-cell leukemia virus 1 infection	KEGG PATHWAY	hsa05166	1	219	0.043973	0.060462	*CANX*

Table S3. GO biological processes analysis of DE lncRNAs co-expression of targeted DE mRNAs (15 terms).

Term	Database	ID	Input number	Background number	*P*-Value	Corrected *P*-Value	Input

protein phosphorylation	Gene Ontology	GO:0006468	15	451	6.12E-07	7.71E-05	*SQSTM1*|*TBK1*|*BUB1B*|*ERCC2*|*SIK2*|*PIK3CD*|*GRK3*|*HSPB8*|*MYLK*|*HIPK3*|*MAPK1*|*CFL1*|*IRAK2*|*DAPK2*|*NRBP1*
positive regulation of NF-kappaB transcription factor activity	Gene Ontology	GO:0051092	9	159	1.98E-06	0.00017554	*TRIM37*|*MAP3K7*|*IL6*|*TFRC*|*CAPN3*|*FLOT1*|*FLOT2*|*IRAK2*|*CLU*
positive regulation of gene expression	Gene Ontology	GO:0010628	12	391	1.79E-05	0.00118926	*UXT*|*TFRC*|*ACTC1*|*PIK3CD*|*WARS*|*IL6*|*MAPK1*|*TNRC6B*|*CLU*|*LMNA*|*CD3E*|*UBR5*
muscle contraction	Gene Ontology	GO:0006936	6	105	9.96E-05	0.003907848	*TNNT3*|*MYH7*|*MYLK*|*TLN1*|*SLMAP*|*TNNI1*
glucose transmembrane transport	Gene Ontology	GO:1904659	3	24	0.000742	0.015709511	*SLC5A10*|*MFSD4B*|*SLC2A12*
positive regulation of smooth muscle cell proliferation	Gene Ontology	GO:0048661	4	58	0.000769	0.01600441	*IL6*|*NR4A3*|*NAMPT*|*STAT1*
cellular response to lipopolysaccharide	Gene Ontology	GO:0071222	6	158	0.000811	0.016444566	*UXT*|*ANKRD1*|*CD274*|*DAB2IP*|*IL6*|*SERPINE1*
skeletal muscle contraction	Gene Ontology	GO:0003009	3	26	0.000917	0.016882827	*TNNT3*|*MYH7*|*TNNI1*
ubiquitin-dependent protein catabolic process	Gene Ontology	GO:0006511	8	292	0.000924	0.016882827	*SQSTM1*|*BUB1B*|*USP45*|*USP14*|*SPSB2*|*RNF34*|*UHRF1*|*PSMC6*
regulation of myoblast differentiation	Gene Ontology	GO:0045661	2	6	0.001186	0.019716713	*CAPN3*|*FLOT2*
activation of MAPK activity	Gene Ontology	GO:0000187	5	123	0.001656	0.023049353	*IRAK2*|*PHB2*|*MAPK1*|*PLCE1*|*MAP3K7*
regulation of striated muscle contraction	Gene Ontology	GO:0006942	2	8	0.001889	0.023559435	*TNNT3*|*TNNI1*
lipid biosynthetic process	Gene Ontology	GO:0008610	2	14	0.004909	0.041627664	*AGPS*|*ACSL1*
regulation of cell population proliferation	Gene Ontology	GO:0042127	5	169	0.006153	0.046561629	*CLU*|*TFRC*|*SQLE*|*STAT1*|*STAT6*
phosphatidylinositol phosphate binding	Gene Ontology	GO:1901981	2	16	0.006204	0.046561629	*RNF34*|*DENND1A*

Table S4. KEGG analysis of DE lncRNAs co-expression of targeted DE mRNAs (15 pathways).

Term	Database	ID	Input number	Background number	*P*-Value	Corrected *P*-Value	Input
AGE-RAGE signaling pathway in diabetic complications	KEGG PATHWAY	hsa04933	6	100	7.71E-05	0.003296	*STAT1*|*PIK3CD*|*IL6*|*MAPK1*|*PLCE1*|*SERPINE1*
Toll-like receptor signaling pathway	KEGG PATHWAY	hsa04620	6	104	9.47E-05	0.003843	*TBK1*|*STAT1*|*PIK3CD*|*MAP3K7*|*IL6*|*MAPK1*
HIF-1 signaling pathway	KEGG PATHWAY	hsa04066	6	109	0.000121	0.004575	*TFRC*|*PFKFB3*|*PIK3CD*|*IL6*|*MAPK1*|*SERPINE1*
Thyroid hormone signaling pathway	KEGG PATHWAY	hsa04919	6	119	0.000191	0.006134	*MYH7*|*STAT1*|*PIK3CD*|*MAPK1*|*PLCE1*|*SIN3A*
NOD-like receptor signaling pathway	KEGG PATHWAY	hsa04621	7	178	0.000245	0.007019	*STAT1*|*TBK1*|*CTSB*|*NAMPT*|*MAP3K7*|*IL6*|*MAPK1*
Osteoclast differentiation	KEGG PATHWAY	hsa04380	6	128	0.000278	0.007837	*SQSTM1*|*STAT1*|*PIK3CD*|*CSF1R*|*MAP3K7*|*MAPK1*
Apoptosis	KEGG PATHWAY	hsa04210	6	136	0.00038	0.009668	*PIK3CD*|*CTSB*|*DAB2IP*|*MAPK1*|*LMNA*|*LAMB2*
Cellular senescence	KEGG PATHWAY	hsa04218	6	160	0.000863	0.016841	*SQSTM1*|*PIK3CD*|*HIPK3*|*IL6*|*MAPK1*|*SERPINE1*
Th17 cell differentiation	KEGG PATHWAY	hsa04659	5	107	0.000914	0.016883	*IL6*|*STAT6*|*MAPK1*|*CD3E*|*STAT1*
Fructose and mannose metabolism	KEGG PATHWAY	hsa00051	3	33	0.001732	0.023432	*ARL1*|*PFKFB3*|*GMDS*
Insulin signaling pathway	KEGG PATHWAY	hsa04910	5	137	0.002604	0.029681	*MAPK1*|*PIK3CD*|*PRKAR1A*|*FLOT2*|*FLOT1*
ECM-receptor interaction	KEGG PATHWAY	hsa04512	4	86	0.003049	0.033334	*AGRN*|*TNXB*|*CD47*|*FRAS1*
IL-17 signaling pathway	KEGG PATHWAY	hsa04657	4	93	0.003989	0.03783	*IL6*|*MAPK1*|*MAP3K7*|*TBK1*
Jak-STAT signaling pathway	KEGG PATHWAY	hsa04630	5	162	0.005187	0.042965	*IL6*|*STAT6*|*STAM*|*PIK3CD*|*STAT1*
T cell receptor signaling pathway	KEGG PATHWAY	hsa04660	4	103	0.005646	0.045355	*MAPK1*|*PIK3CD*|*CD3E*|*MAP3K7*

Table S5. The primers used for the validation of lncRNAs and mRNAs.

Primer Name	Sequence(5'to3')	Tm/°C	Product Size/bp
GAPDH-F	GCCAAAAGGGTCATCATCTC	53.6	287
GAPDH-R	GTAGAGGCAGGGATGATGTTC	55.2	
ZNF148-F	AGTCGGTGCTTCAAGATCGG	60.11	132
ZNF148-R	TCACTGTCTCCTCATGGACC	58.44	
STAT6-F	CAGCCCAGATATGGTGTCCC	59.89	108
STAT6-R	TCCTGGAAGGCTGACAACAC	59.89	
ACTB-F	CTCTTCCAGCCCTCCTTCCT	59.32	177
ACTB-R	GGCCGTGATCTCCTTCTGCAT	60.25	
CMYA5-F	GCCACTGAGACCTACACT	53.83	175
CMYA5-R	CTTCACTCTGTTCACTTGTC	50.35	
FHOD3-F	TTCCACCTCTTCTCCTATGA	51.58	137
FHOD3-R	CGGAAGTTGTAAGGTTCAGA	51.58	
LNC_000040-F	CGAATCTCCTCGTCCTGCTC	58.05	158
LNC_000040-R	ATTGTCCCAAGCAGCGGTAA	57.05	
LNC_004737-F	GCAAGTGCATTAAGGGCTCG	57.26	169
LNC_004737-R	TGGGTTCATTAAGCTCGCGT	56.95	
LNC_006333-F	ACGGGTACTGGCATTTGGTT	57.06	196
LNC_006333-R	AAGGGAAAGTTGGGCACACA	57.28	
LNC_008264-F	TTCTAATTCGCAACCTGAGA	50.43	163

LNC_008264-R	CAAGCCCTAGTAGCCATTT	51.73	
LNC_002605-F	GCCTCTGATTCACTTGACT	51.58	205
LNC_002605-R	AATGCTGCTGGACTGTTC	52.67	
